# Early-life factors are associated with waist circumference and type 2 diabetes among Ghanaian adults: The RODAM Study

**DOI:** 10.1038/s41598-019-47169-6

**Published:** 2019-07-26

**Authors:** Ina Danquah, Juliet Addo, Daniel Boateng, Kerstin Klipstein-Grobusch, Karlijn Meeks, Cecilia Galbete, Erik Beune, Silver Bahendeka, Joachim Spranger, Frank P. Mockenhaupt, Karien Stronks, Charles Agyemang, Matthias B. Schulze, Liam Smeeth

**Affiliations:** 1 Department of Molecular Epidemiology, German Institute of Human Nutrition Potsdam-Rehbruecke (DIfE), Nuthetal, Germany; 2 Charité – Universitaetsmedizin Berlin, corporate member of Freie Universität Berlin, Humboldt-Universität zu Berlin, and Berlin Institute of Health, Institute for Social Medicine, Epidemiology and Health Economics, Berlin, Germany; 3 Department of Non-Communicable Disease Epidemiology, Faculty of Epidemiology and Population Health, London School of Hygiene and Tropical Medicine, London, United Kingdom; 4 Julius Global Health, Julius Center for Health Sciences and Primary Care, University Medical Center Utrecht, Utrecht University, Utrecht, The Netherlands; 5 Division of Epidemiology & Biostatistics, School of Public Health, Faculty of Health Sciences, University of the Witwatersrand, Johannesburg, South Africa; 6 Department of Public Health, Amsterdam Public Health Research Institute, Academic Medical Center, Amsterdam UMC, University of Amsterdam, Amsterdam, The Netherlands; 7 Mother Kevin Postgraduate Medical School (MKPGMS), Uganda Martyrs University, Kampala, Uganda; 8 Charité – Universitaetsmedizin Berlin, corporate member of Freie Universitaet Berlin & Humboldt-Universitaet zu Berlin, and Berlin Institute of Health, Department of Endocrinology and Metabolism; DZHK (German Centre for Cardiovascular Research), partner site Berlin; Center for Cardiovascular Research (CCR), Berlin, Germany; 9 Charité – Universitaetsmedizin Berlin, corporate member of Freie Universitaet Berlin & Humboldt-Universitaet zu Berlin, and Berlin Institute of Health, Institute of Tropical Medicine and International Health, Berlin, Germany

**Keywords:** Epidemiology, Risk factors

## Abstract

Early-life experiences may fuel the emergence of obesity and type 2 diabetes among African populations. We evaluated childhood socio-economic status (SES) and childhood nutritional status as risk factors for increased waist circumference and type 2 diabetes among Ghanaian adults. In the multi-center, cross-sectional Research on Obesity and Diabetes among African Migrants (RODAM) Study, we calculated associations (adjusted for demographics and lifestyle) of parental education and anthropometric markers of childhood nutrition [leg length, leg length-to-height ratio (LHR)] with waist circumference and type 2 diabetes, respectively. Among 5,575 participants (mean age: 46.2 years; 62% female), lower education of either parent (vs. higher) was consistently associated with higher waist circumference (∆: 1.6–3.4 cm). Lower father’s education tended to increase the odds of type 2 diabetes by 50% in women (95% confidence interval (CI): 1.0, 2.4). Reduced leg length and LHR were associated with higher waist circumference. But only in men, leg length was inversely related to type 2 diabetes (OR per 1 standard deviation decrease: 1.1; 95% CI: 1.0, 1.3). In this study, markers of poor childhood SES and early-life nutritional status relate to abdominal obesity in men and women and to type 2 diabetes in men. Thus, prevention efforts should start in early childhood.

## Introduction

Obesity and type 2 diabetes are important public health issues globally^1,2^. There is accumulating evidence of the increasing burden of both obesity and type 2 diabetes in sub-Saharan Africa (SSA). Yet, the prevalence rates vary considerably between adults in SSA and among SSA migrants^3^. Increased life expectancy, rapid urbanization and its associated lifestyle changes are potential drivers of the upsurge of obesity and type 2 diabetes in SSA^4^. Still, undernutrition continues to plague both children and adults in SSA, while the co-existence with overnutrition has been described for the same settings^5^. In fact, the abundance of undernutrition and the rapid environmental changes in SSA and for first-generation migrants from Africa to Europe might constitute one of the underlying causes of the emergence of metabolic conditions in these populations. There is evidence from high-income countries linking early-life conditions and childhood growth patterns to the pathogenesis of chronic diseases^6,7^. Low childhood socioeconomic status (SES) and undernutrition during early development have been shown to be associated with an increased risk of obesity and type 2 diabetes^8,9^. Still, the evidence is scarce and inconsistent for African-descent populations^10,11^.

According to the mismatch hypothesis within the Developmental Origins of Health and Disease (DOHaD) framework, a deprived developmental environment sets up an individual’s metabolism to nutrient scarcity. This programming effect becomes detrimental when the mature environment is characterized by nutritional abundance^12^. Exploring the associations of childhood SES and undernutrition with obesity and type 2 diabetes could offer insights into the increasing burden of these conditions in SSA.

Therefore, we aimed to assess whether childhood SES and childhood nutritional status are associated with abdominal obesity and type 2 diabetes in adulthood, independently of adult SES in a homogenous population of Ghanaians living in different geographical locations (rural Ghana, urban Ghana and Europe). We hypothesized that adverse pre-adulthood social environments measured by parental education and anthropometric markers of childhood undernutrition (defined as leg length and leg length-to-height ratio) are associated with increased risks of abdominal obesity and type 2 diabetes, and that the associations are independent of adult lifestyle and SES.

## Methods

### Study design and procedures

The study protocol and procedures of the Research on Obesity and Diabetes among African Migrants (RODAM) study have previously been published^13^. In brief, this multi-center cross-sectional study was conducted among Ghanaian adults (25–70 years) in rural Ghana, urban Ghana, and Europe (Amsterdam, London, and Berlin) between July 2012 and September 2015 (N = 6,385). The primary objective of the RODAM study was to disentangle the relative contributions of (epi)genetic and non-genetic risk factors for type 2 diabetes and obesity. For recruitment, in Ghana, census data of 2010 were used to draw rural and urban participants in the Ashanti Region. In Amsterdam, the Municipal Register was used to randomly select Ghanaian migrants who were then invited by postal mail and home visits. In London and Berlin, Ghanaian organizations, church communities and social unions served as the sampling frame for recruitment. The response rates were 76% in rural Ghana and 74% in urban Ghana. In Amsterdam, 67% replied by response card or after a home visit. Of these, 53% agreed and participated in the study. In London, of those individuals who were invited based on their registration in Ghanaian organizations, 75% agreed and participated in the study. In Berlin, this figure was 68%^3^.

Fasting plasma glucose (FPG; mmol/L) was measured in venous blood samples using the ABX Pentra 400 chemistry analyzer (HORIBA ABX SAS, Montpellier, France). Type 2 diabetes was defined as FPG ≥7.0 mmol/L or documented glucose-lowering medication or self-reported diabetes. Medical history, lifestyle and socio-economic factors were recorded either by ethnically matched staff in questionnaire-based interviews or by self-report. Trained study personnel performed the physical examination, including anthropometric measurements.

### Ethics statement

The RODAM Study was conducted according to the guidelines laid down in the 1964 Declaration of Helsinki and its later amendments. All procedures involving human subjects were reviewed and approved by the respective ethics committees in Ghana (Committee on Human Research, Publication and Ethics, Kwame Nkrumah University of Science and Technology, Kumasi), the Netherlands (Medical Ethics Review Committee, Academic Medical Centre, University of Amsterdam), the UK (Observational/Interventions Research Ethics Committee, London School of Hygiene and Tropical Medicine), and Germany (Ethics Commission, Charité – Universitaetsmedizin Berlin). Written informed consent was obtained from all participants.

### Questionnaire-based data

The educational levels of the participants and their parents were recorded according to the following categories: never been to school or elementary school, lower vocational schooling, i.e. <2 years training or lower secondary schooling, intermediate vocational schooling, i.e. 2 years training or intermediate/higher secondary schooling, and higher vocational schooling, i.e. ≥3 years training or university. Occupational class was recorded for the participants only and was classified as “high” (professionals, managers, clerical support staff, higher grade routine non-manual employees service and sales related occupations) and “low” (craft and related trades workers, elementary occupations and farmers) (International Standard Classification of Occupations scheme; ISCO-08). In addition, sociodemographic (age and sex) and lifestyle factors were obtained. Smoking status was recorded as current, former or never. Physical activity was assessed using the World Health Organization (WHO) STEPwise approach to chronic disease risk factor Surveillance (STEPS) Questionnaire and was categorized as high, moderate, or low. Energy intake was calculated from a semi-quantitative Ghana-specific Food Propensity Questionnaire (Ghana-FPQ) which queried for the usual food intake of the preceding 12 months. The latest versions of the West African Food Composition Table and the German Nutrient Database (BLS 3.01, 2010) were applied to convert food intake data into energy intake.

### Anthropometric examinations

All participants underwent an anthropometric examination in light clothing and without shoes. Body weight (kg) was measured using a portable electronic scale (SECA 877) to the nearest 0.1 kg, height (cm) by a portable stadiometer (SECA 217) to the nearest 0.1 cm, and waist circumference (cm) measured at the at the midpoint between the lower rib and the upper margin of the iliac crest using a measuring tape. Body mass index (BMI) was calculated as weight/(height)^2^ (kg/m^2^). Abdominal obesity was defined as waist circumference >102 cm for men, and >88 cm for women. Adult leg length is particularly sensitive to nutrition in early childhood^14^. Therefore, we measured sitting height with the participant sitting upright on the base plate on a flat seat, and with the heads in the Frankfort plane position, feet on the floor and with the thighs unsupported. Leg length (cm) was calculated as (standing height − sitting height) + stool height, while leg length-to-height ratio (LHR) was calculated as leg length divided by standing height^15^. Fat-free mass was examined using bio-electric impedance (BODYSTAT 1500 MDD analyser) with an African-specific equation.

### Statistical analysis

Supplemental Fig. S1 shows the flow chart of imputed raw data and excluded participants because of implausible values for composite measures yielding a final sample size of 5,575 participants. A small proportion had missing values for waist circumference (0.3%), but a considerable proportion had missings for leg length, height and parental education (24%), and for the socio-demographic and lifestyle factors (45%). Excluding these participants might have led to biased results and a loss in statistical power. Therefore, multiple imputation was applied (n = 5; discriminant fully conditional specification (FCS) method). FCS is also known as multiple imputation by chained equations (MICE) and uses separate conditional univariate imputation models specified for each incomplete variable, with other variables as predictors^16^. We assumed that the propensity of missing data can be explained by the observed data (missing at random, MAR), because the proportions of missingness were similar between the groups with and without type 2 diabetes (Fig. S1). This assumption was further corroborated by the good imputation efficiency (98–100%).

Descriptive statistics were used to present the general characteristics of the study population and the distributions of early-life factors according to sex and study site.

Paternal education and maternal education were categorized as never/low, intermediate, and high; we used high education as the reference. Relationships between parental education and the adult educational level and occupational class were calculated by partial Spearman correlations, adjusted for age and study site. For leg length and LHR, sex-specific quintiles were constructed, and the highest category was used as the reference. Among Ghanaians, multiple parenthood and child fostering are common practices^17^. Therefore, we did not assume that the combination of father’s and mother’s educational levels reflected the true SES during childhood.

All regression models were calculated separately for men and women owing to their differences in body fat distribution and adipose tissue function^18^. With regard to abdominal obesity, we modeled waist circumference as a transient phenotype and thus, as a continuous variable. The PROC MIANALYZE procedure in SAS was used to calculate sex-specific pooled ß-coefficients, 95% confidence intervals (CIs) and p-values for the associations of parental education with waist circumference by linear regressions. Also, the associations of leg length and LHR with waist circumference were evaluated per 1 standard deviation (SD) decrease using pooled effect estimates of linear regression models. Model 1 accounted for age (years) and study site (5 categories); Model 2 was additionally adjusted for the most important lifestyle risk factors of adiposity and type 2 diabetes: smoking (current or former/non-smoker), physical activity (MET-h/week), and energy intake (kcal/d). For leg length, Model 3 additionally accounted for body height (cm).

With respect to type 2 diabetes, we calculated sex-specific pooled odds ratios (ORs), 95% CIs and p-values by logistic regression models. Model 1 was adjusted for age and study site; and Model 2 additionally accounted for smoking status, physical activity, energy intake, BMI and waist circumference. The associations with type 2 diabetes were calculated per quintiles and per 1 SD decrease of leg length and LHR, respectively.

Despite the lack of statistically significant interactions with study site, we calculated multiple-adjusted associations of leg length and LHR with waist circumference and with type 2 diabetes according to study site to comment on potential effect modification. Because of sample size constrains, this was not done for parental education. Complete-case analysis was performed to examine the robustness of our results. Also, we evaluated effect mediation: Adult socio-economic status *per se* has been related to higher odds of type 2 diabetes in the RODAM study population^19^, and thus, could lie on the causal pathway from parental education in childhood to higher waist circumference and increased odds of type 2 diabetes in adulthood. Similarly, childhood nutritional status may act *via* fat-free mass on the adult metabolic profile, whereby fat-free mass reflects the attained metabolic capacity of muscle mass^20^. These proposed mediators were included in the final regression models to assess effect attenuation. Moreover, we assessed potential confounding: by diabetes family history when parental education was examined, and by hip circumference when leg length was considered. Potential changes in the effect estimates were examined by including likely confounders in the final regression models.

## Results

### Study population

The general characteristics of the study population according to sex and study site are presented in Supplemental Table S1. In brief, RODAM participants were mainly female (62%) and middle-aged (mean age, 46.2 ± 10.8 years). The mean waist circumference in the study population was 90.7 ± 12.6 cm, and was higher in women than in men. The crude prevalence of type 2 diabetes was 9.4%. This figure was lower in women than in men and was highest in Europe, followed by urban Ghana and rural Ghana. Men were older, had a higher educational status, had lower BMI and waist circumference, were more likely to be former or current smokers, were more physically active, and had higher daily energy intake than women. RODAM participants in Europe had the highest level of education, were more frequently former or current smokers, presented with higher BMI and waist circumference, and men in Europe had higher energy consumption than their counterparts in Ghana. The mean length of stay in Europe was 16.9 ± 9.9 years, and 99% were first-generation migrants. RODAM participants in rural Ghana had the lowest level of education, the lowest BMI and waist circumference, and were physically more active than those in urban Ghana and Europe.

With regard to the early-life factors (Table 1), the parental education was similar for men and women. Father’s and mother’s education were lowest for the participants living in rural Ghana and was highest in Europe. For leg length and LHR, men had longer legs than women. Leg length among men was lowest in rural Ghana, followed by urban Ghana and Europe, while no differences according to study site were discernible among women. For men, partial Spearman correlations of parental education with adult education ranged from +0.27 to +0.31 (p < 0.0001) and with adult occupation from −0.21 to −0.19 (p < 0.0001). For women, these figures were +0.27 to +0.34 (p < 0.0001) and −0.16 to −0.16 (p < 0.0001), respectively.

**Table 1:** Distributions of early-life factors according to sex and study site.

Characteristics	Total (N = 5,575)	Rural Ghana (n = 1,004)	Urban Ghana (n = 1,440)	Europe (n = 3,131)
**Male (n)**	**2,118**	**397**	**409**	**1,312**
Age (years)	46.8 ± 11.1	46.3 ± 12.9	46.6 ± 11.9	47.1 ± 10.3
Father’s education				
Never or elementary	58.5%	82.3%	63.4%	49.8%
Low	13.3%	10.2%	22.4%	11.4%
Intermediate	16.5%	4.8%	9.2%	22.3%
Higher vocational	11.7%	2.7%	4.9%	16.5%
Mother’s education				
Never or elementary	74.7%	89.3%	81.9%	68.1%
Low	9.9%	6.9%	13.4%	9.8%
Intermediate	7.3%	1.8%	2.9%	10.4%
Higher vocational	8.0%	2.0%	1.9%	11.8%
Waist circumference (cm)	88.1 ± 12.0	76.7 ± 8.1	84.7 ± 10.5	92.5 ± 10.8
Height (cm)	170.8 ± 6.8	168.7 ± 7.3	169.6 ± 6.9	171.8 ± 6.4
Leg length (cm)	86.0 ± 4.4	85.3 ± 4.6	85.8 ± 4.5	86.3 ± 4.4
Quintile 1 of leg length (<82.1 cm)	20.2%	26.4%	23.2%	17.3%
Leg length-to-height ratio	0.50 ± 0.01	0.51 ± 0.01	0.51 ± 0.01	0.50 ± 0.01
Quintile 1 of leg length-to-height ratio (<0.49)	19.9%	13.1%	16.3%	23.1%
**Female (n)**	**3,457**	**607**	**1,031**	**1,819**
Age (years)	45.8 ± 10.6	46.6 ± 12.4	44.7 ± 11.2	46.2 ± 9.6
Father’s education				
Never or elementary	57.1%	83.7%	64.9%	43.8%
Low	15.5%	10.2%	22.5%	13.2%
Intermediate	16.3%	3.5%	8.5%	25.0%
Higher vocational	11.2%	2.6%	4.2%	18.0%
Mother’s education				
Never or elementary	76.5%	91.3%	84.6%	67.0%
Low	10.3%	6.4%	11.5%	10.8%
Intermediate	6.6%	1.2%	2.7%	10.7%
Higher vocational	6.6%	1.2%	1.1%	11.5%
Waist circumference (cm)	92.3 ± 12.7	83.7 ± 11.3	91.2 ± 11.9	95.9 ± 12.1
Height (cm)	159.9 ± 6.2	157.8 ± 6.4	158.7 ± 5.9	161.2 ± 6.0
Leg length (cm)	80.0 ± 4.1	79.7 ± 4.1	80.1 ± 4.1	80.1 ± 4.1
Quintile 1 of leg length (<76.4 cm)	20.1%	21.9%	18.6%	20.3%
Leg length: height	0.50 ± 0.01	0.50 ± 0.01	0.50 ± 0.01	0.50 ± 0.01
Quintile 1 of leg length-to-height ratio (<0.48)	20.0%	9.3%	10.1%	29.2%

Data are presented as mean ± standard deviation and as percentage.

### Associations with parental education

Table 2 presents the associations of parental education with waist circumference and with type 2 diabetes, separately for men and women. In general, the educational level of the parents was inversely associated with waist circumference. For instance, in men, lower paternal education as compared to higher was associated with an increased waist circumference of 3.6 cm (95% CI: 2.1, 5.1 cm) when adjusted for age and study site. This association was also seen for maternal education and remained in the lifestyle-adjusted Model 2. For women, these inverse relationships between parental education and waist circumference were also observed (Table 2).

**Table 2:** Multiple-adjusted associations of parental education with waist circumference by sex.

Waist circumference	Men				Women			
	Model 1 (β; 95% CI)	p	Model 2 (β; 95% CI)	p	Model 1 (β; 95% CI)	p	Model 2 (β; 95% CI)	p
Father’s education								
Never/low	3.6 (2.1, 5.1)	<0.0001	3.4 (1.9, 4.9)	<0.0001	1.7 (0.2, 3.1)	0.022	1.6 (0.2, 3.0)	0.022
Intermediate	2.6 (1.0, 4.1)	0.002	2.4 (0.9, 4.0)	0.003	1.8 (0.62, 3.0)	0.003	1.9 (0.7, 3.1)	0.002
Higher	Reference		Reference		Reference		Reference	
Mother’s education								
Never/low	3.4 (1.7, 5.2)	0.0002	3.1 (1.4, 4.9)	0.0004	1.9 (0.1, 3.7)	0.042	1.9 (0.1, 3.6)	0.043
Intermediate	2.8 (0.4, 5.1)	0.025	2.5 (0.2, 4.9)	0.036	−0.6 (−2.3, 1.1)	0.488	−0.6 (−2.4, 1.1)	0.474
Higher	Reference		Reference		Reference		Reference	
**Type 2 diabetes**	**Men**				**Women**			
	**Model 1** **(OR; 95% CI)**	**p**	**Model 2** **(OR; 95% CI)**	**p**	**Model 1** **(OR; 95% CI)**	**p**	**Model 2** **(OR; 95% CI)**	**p**
Father’s education								
Never/low	0.86 (0.51, 1.46)	0.578	0.74 (0.43, 1.30)	0.295	1.36 (0.89, 2.08)	0.147	1.50 (0.96, 2.36)	0.077
Intermediate	1.06 (0.65, 1.71)	0.818	0.94 (0.58, 1.53)	0.806	0.85 (0.57, 1.28)	0.446	0.86 (0.56, 1.32)	0.494
Higher	Reference		Reference		Reference		Reference	
Mother’s education								
Never/low	0.76 (0.40, 1.45)	0.409	0.65 (0.34, 1.27)	0.210	0.91 (0.49, 1.71)	0.772	0.93 (0.48, 1.78)	0.823
Intermediate	1.21 (0.55, 2.66)	0.627	1.06 (0.48, 2.33)	0.890	0.70 (0.34, 1.45)	0.335	0.75 (0.35, 1.58)	0.441
Higher	Reference		Reference		Reference		Reference	

For waist circumference (upper part of the table), beta-coefficients (β), 95% confidence intervals (CIs) and p-values were calculated by linear regression. For type 2 diabetes (bottom part of the table), odds ratios, 95% CIs and p-values were calculated by logistic regression. Model 1 accounted for age (years) and study site (5 categories). Model 2 was additionally adjusted for smoking (current or quit/never), physical activity (MET-hours/week), and energy intake (kcal/d), and for waist circumference (cm) in the logistic model.

For type 2 diabetes, among men, neither father’s nor mother’s education were associated with this outcome. In contrast, among women, lower father’s education as compared to higher education tended to increase the odds of type 2 diabetes by 50% (1.50; 95% CI: 0.96, 2.36) in the multiple-adjusted Model 2. Otherwise, parental education was not associated with type 2 diabetes among women. Owing to sample size constraints, no further stratification by study site was performed.

### Associations with leg length and leg length-to-height ratio

The sex-specific associations of leg length and LHR with waist circumference are presented per quintiles and per 1 SD decrease in Table 3. Absolute leg length was directly associated with waist circumference in Models 1 and 2 among men and women. However, these effects reversed after accounting for body height in Model 3. In men, 1 SD reduction in leg length (1 SD = 4.4 cm) increased waist circumference by 1.72 cm (95% CI: 0.89, 2.54 cm). In women (1 SD = 4.1 cm), this increment was 2.42 cm (95% CI: 1.64, 3.20 cm). Also, LHR was associated with higher waist circumference in men and women (Table 3). In men, 1 SD reduction in LHR resulted in an increase of 0.50 cm (95% CI: 0.05, 0.95 cm), while in women this effect was 0.76 cm (95% CI: 0.33, 1.18 cm). The inverse associations of leg length and LHR were strongest in urban Ghana (Supplemental Table S2).

**Table 3:** Multiple-adjusted associations of leg length (cm) and leg length-to-height ratio with waist circumference by sex.

Leg length (cm)	β coefficient (95% confidence interval)					p trend	β per 1 SD decrease (95% CI)	p
	Q1	Q2	Q3	Q4	Q5			
**Leg length (cm)**								
**Men**								
Waist circumference (cm)	86.6 ± 13.3	87.7 ± 11.3	88.0 ± 12.0	88.3 ± 11.8	89.8 ± 11.5			
Model 1	−3.88 (−5.27, −2.49)	−2.96 (−4.35, −1.57)	−2.15 (−3.55, −0.76)	−1.72 (−3.10, −0.33)	1.00	<0.0001	−1.36 (−1.80, −0.91)	<0.0001
Model 2	−3.87 (−5.25, −2.48)	−2.99 (−4.38, −1.61)	−2.13 (−3.52, −0.75)	−1.73 (−3.11, −0.35)	1.00	<0.0001	−1.35 (−1.79, −0.92)	<0.0001
Model 3	4.05 (1.77, 6.33)	2.42 (0.58, 4.26)	1.79 (0.15, 3.42)	0.84 (−0.64, 2.32)	1.00	0.0003	1.72 (0.89, 2.55)	<0.0001
**Women**								
Waist circumference (cm)	91.3 ± 12.6	91.7 ± 11.9	91.8 ± 13.6	93.2 ± 12.3	93.7 ± 12.9			
Model 1	−2.89 (−4.14, −1.64)	−2.31 (−3.57, −1.05)	−2.10 (−3.38, −0.83)	−0.73 (−1.99, 0.53)	1.00	<0.0001	−1.10 (−1.49, −0.70)	<0.0001
Model 2	−2.90 (−4.15, −1.65)	−2.31 (−3.57, −1.06)	−2.13 (−3.40, −0.86)	−0.76 (−2.02, 0.50)	1.00	<0.0001	−1.10 (−1.49, −0.70)	<0.0001
Model 3	5.41 (3.36, 7.47)	3.55 (1.86, 5.24)	2.23 (0.73, 3.74)	2.07 (0.71, 3.42)	1.00	<0.0001	2.42 (1.65, 3.20)	<0.0001
**Leg length-to-height ratio**								
**Men**								
Waist circumference (cm)	91.0 ± 12.2	88.4 ± 12.1	87.7 ± 12.3	87.1 ± 11.9	86.2 ± 11.1			
Model 1	1.81 (0.39, 3.23)	0.89 (−0.52, 2.30)	0.91 (−0.49, 2.30)	0.76 (−0.64, 2.15)	1.00	0.020	0.52 (0.07, 0.97)	0.024
Model 2	1.75 (0.33, 3.17)	0.90 (−0.50, 2.30)	0.88 (−0.51, 2.27)	0.77 (−0.61, 2.16)	1.00	0.022	0.50 (0.05, 0.95)	0.030
**Women**								
Waist circumference (cm)	94.9 ± 13.0	92.9 ± 11.7	92.3 ± 13.1	91.3 ± 12.5	90.4 ± 12.7			
Model 1	1.91 (0.57, 3.24)	1.49 (0.21, 2.77)	1.51 (0.23, 2.80)	0.57 (−0.71, 1.85)	1.00	0.002	0.75 (0.32, 1.17)	0.0006
Model 2	1.92 (0.59, 3.26)	1.45 (0.17, 2.73)	1.54 (0.26, 2.82)	0.55 (−0.73, 1.82)	1.00	0.002	0.76 (0.33, 1.18)	0.0005

Waist circumference is presented as mean ± standard deviation. Beta-coefficients (β), 95% confidence intervals (CIs) and p-values for waist circumference were calculated by linear regression. Model 1 accounted for age (years) and study site (5 categories). Model 2 was additionally adjusted for smoking (current or quit/never), physical activity (MET-hours/week), and energy intake (kcal/d). Model 3 was additionally adjusted for body height (cm).

**Figure 1:**
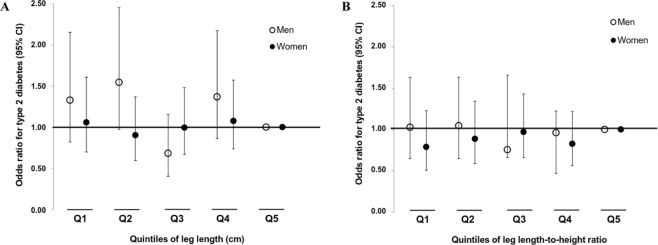
Multiple-adjusted associations of (**A**) leg length (cm) and (**B**) leg length-to-height ratio with type 2 diabetes stratified by sex. Odds ratios and 95% confidence intervals (error bars) for type 2 diabetes were calculated by logistic regression and were adjusted for age (years), study site (5 categories), smoking (current or quit/never), physical activity (MET-hours/week), energy intake (kcal/d), body mass index (kg/m^2^), and waist circumference (cm).

With regard to type 2 diabetes, the associations per quintiles of leg length and LHR are depicted in Fig. 1. Among men, lower quintiles tended to increase the odds of type 2 diabetes in the multiple-adjusted Model 2 (OR per 1 SD leg length decrease: 1.11; 95% CI: 0.95, 1.30; p = 0.186). However, quintile 3 showed the lowest odds of type 2 diabetes. Further, we explored the associations between absolute and relative leg lengths according to study location. The inverse tendency for the LHR-diabetes-relationship was strongest in rural Ghana (Table S2), but was neither seen for women nor for LHR (Fig. 1).

### Sensitivity analyses

Compared to the results from imputed data, the associations in a complete-case analysis became stronger in men and weaker in women for waist circumference. For type 2 diabetes, there were no major changes (Supplemental Table S3). With regard to adult socio-economic variables, we evaluated whether adult education or adult occupation mediated the observed relationships with parental education (Supplemental Table S4). For waist circumference, the associations were attenuated by adult education and adult occupation. The changes in estimates were slightly stronger when accounting for adult education. Next, the inverse association between father’s education and type 2 diabetes in women attenuated from OR = 1.5 (95% CI: 1.0, 2.4) to OR = 1.4 (95% CI: 0.9, 2.2). Otherwise, the effect estimates for parental education on type 2 diabetes remained. Also, the inclusion of diabetes family history did not alter the relationships between parental education and diabetes status (data not shown). Lastly, for the associations of leg length and LHR with type 2 diabetes, we evaluated potential effect mediation by fat-free mass and adult SES as well as possible confounding by hip circumference and parental education. Yet, none of these factors changed the estimates (data not shown).

## Discussion

### Summary of main results

The present study investigated the mismatch hypothesis of adverse early-life factors conferring increased risks of abdominal obesity and type 2 diabetes among Ghanaian adults facing rapid environmental changes. Indeed, low childhood SES defined as low education of one parent was consistently associated with higher waist circumference among men and women. Partly, these effects were explained by the fact that low parental education was associated with lower adult educational level and lower adult occupational class, which in turn were related to higher waist circumference and type 2 diabetes risk^19^. Also, there was a tendency for increased odds of type 2 diabetes in women when the education of the father was lower. Moreover, childhood undernutrition defined as low absolute and relative leg lengths tended to be associated with higher odds of type 2 diabetes in men and was associated with higher waist circumference in both sexes. These relationships were more pronounced in Ghana than in Europe and were largely independent of parental education.

### Associations of parental education with waist circumference and type 2 diabetes

The inverse association between parental education and waist circumference was observed in both men and women, although stronger in men. The association was partly explained by the participant’s own educational attainment and occupation. This finding is consistent with that from previous studies which have reported a higher risk of CVD and its associated factors such as obesity and type 2 diabetes in men and women with lower childhood SES^21,22^. In a large cohort of British children studied from birth to 36 years, men and women from lower childhood socioeconomic backgrounds were more inclined to be overweight or obese in adulthood^23^. Adult SES has partly explained the observed association in some but not all previous studies^24–26^. Several mechanisms underlying these observations independent of access to health care have been proposed, including physical, environmental, behavioral and psychosocial exposures^21,22^. Lower SES in childhood has been reportedly associated with diseases caused largely by behavioral risk factors such as smoking, physical inactivity and unhealthy diets^27^. Possibly, children of lower SES are more likely to live in homes and neighborhoods less conducive for engaging in physical activity, placing them at higher risk of childhood obesity which may set health-damaging trajectories that continue through adulthood^21^.

There was no clear association between childhood SES and type 2 diabetes in men in our study, although there was a tendency for increased odds of type 2 diabetes in women from a lower childhood SES, independent of current BMI and other risk factors. This was contrary to the inverse association reported in some^28^ but not all previous studies^29^. Lower paternal education has been reported to be more obviously associated with type 2 diabetes in women than in men in a previous study^29^. It remains unclear why the association between childhood SES and type 2 diabetes was not as obvious in our study, given the observed association with waist circumference.

### Associations of leg length with waist circumference and type 2 diabetes

In the present study, absolute and relative leg lengths were associated with higher adult waist circumference in men and women and tended to increase the odds of type 2 diabetes in men. Among white populations living in high-income countries, shorter legs and lower LHR are consistently associated with higher measures of adiposity and diabetes-risk, including the PROMISE cohort in Canada as well as NHANES III and ARIC in the USA^11,20,30,31^. Still, the evidence has been scarce and inconclusive for individuals of African descent and for populations in less-developed regions, such as China and Brazil^10,11,30,32,33^.

Body height *per se* is determined by genetic and environmental factors, whereas the anthropometric measures of disproportion such as leg length and LHR sensitively indicate postnatal childhood nutritional status^15^. The results of the present study are therefore in accordance with the hypothesis that individuals are prone to develop cardio-metabolic diseases when a nutrient-insufficient developmental environment conflicts with nutrient surplus in the future^12^. Indeed, Ghana experienced three major droughts in 1971, 1977 and 1983^34^, in addition to a period of military rule and economic crisis between 1964 and 1992. For the present study population of Ghanaian adults aged ≥35 years, this means that the majority were exposed to at least one period of famine in their childhood. After that, in the last decades, Ghana’s economy has rapidly improved. The Gross Domestic Product (GDP) has increased from US$ 4.5 billion in 1980 to US$ 42.7 billion in 2016, and more than half of Ghana’s population today lives in large cities^35^. Such economic boost and the sudden environmental change upon urbanization and migration to Europe might have led to a nutrition transition, as observed across the RODAM study sites. In rural Ghanaian sites, the diet relies on tuberous foods, legumes and fish, while in urban Ghana and Europe, ruminant meat, dairy products, sweet snacks and energy-containing beverages predominate^36^. Partially, this argues for a pronounced mismatch between the developmental and the mature environments among urban Ghanaians and migrants in Europe, corroborating the stronger inverse associations of leg length and LHR with waist circumference as compared to rural Ghana.

The biological mechanisms underlying the increased susceptibility of abdominal obesity and type 2 diabetes in this transitional society may include the accumulation of fat mass upon chronic childhood undernutrition^14^, possibly, because of impaired appetite control^37^ and reduced fat oxidation^38^. Also, delayed growth in childhood contributes to altered organ development and reduced skeletal muscle mass, resulting in impaired pancreatic function and reduced peripheral insulin sensitivity, respectively^39,40^. However, in our study, adjustment for fat-free mass as a measure of skeletal muscle mass did not contribute to the association between leg length and type 2 diabetes.

In addition, there was a lack of association for absolute and relative leg lengths with type 2 diabetes among women in our study. Smaller variations in leg length and LHR, and a lower prevalence of type 2 diabetes among females (8%) as compared to males (11%) could explain this null-finding. It is also conceivable that selective survival might have affected our results. The vicious circle of poor childhood nutritional status and infectious diseases still leads to a mortality rate of 5.9% among children <5 years of age in Ghana^35^, presumably attenuating the leg length-diabetes association in adulthood.

### Strengths and limitations

The present analysis stems from a large, homogenous study population of SSA adults living in Ghana and in Europe. The participants originated from the same geographic region in Ghana and likely were exposed to the historic waves of famine and food crisis in the country. Still, we cannot fully exclude the positive selection of more affluent individuals migrating to Europe and the negative selection of people who experienced severe childhood undernutrition and illness. Multiple imputation was applied for an unbiased analysis of the full dataset. Under the MAR assumption, this method is useful even when 90% of the data are missing^41^. In our regression models, we accounted for a wide range of possible confounders and potential mediators. Still, unmeasured and residual confounding cannot be ruled out. Similar to most previous studies examining early-life factors on adult health, our study included assessment of only one SES marker. We did not take into account other components of SES that could potentially have an impact on health such as household income and living conditions during childhood, as well as SES across the entire life-course. Previously, the recollection of parental education has less likely been associated with recall bias^41,42^. We are aware of the genetic and the socio-economic components of attained height and leg length, and adjusted for parental education in our sensitivity analyses. Still, relative leg length represents a widely accepted and objective marker of early childhood nutritional status^15^. While the cross-sectional nature of our study impedes causal inference and the investigation of cumulative effects across the life-course, the exposures of interest were likely established before the onset of metabolic disturbances in adulthood.

## Conclusions

Interventions to prevent abdominal obesity may have to consider targeting the early childhood period and adults who had experienced lower SES in childhood. Further studies examining the association of early-life socioeconomic and nutritional factors with waist circumference and type 2 diabetes are needed in lower- and middle-income populations. This is of particular importance in the light of the reported increasing burden of abdominal obesity and type 2 diabetes, emerging alongside an unfinished agenda of undernutrition and other poverty related diseases in these settings. Our findings emphasize that targeted improvement of early-life circumstances can have benefits not just for children but also in reducing the risks of metabolic disease in later adult life.

## Supplementary information

Supplementary information

## Data Availability

The datasets analysed during the current study are not publicly available due to the terms of consent to which the participants agreed, but are available from the corresponding author on reasonable request.
