# Supplementary material for: Early-life factors are associated with waist circumference and type 2 diabetes among Ghanaian adults: The RODAM Study
Source: Sci Rep. 2019 Jul 26;9:10848. doi: 10.1038/s41598-019-47169-6 (PMC6659619; doi:10.1038/s41598-019-47169-6)
Supplement: Supplementary file 1 — Supplementary information [file 41598_2019_47169_MOESM1_ESM.docx]

**Supplementary information**

**Title:** Early-life factors are associated with waist circumference and type 2 diabetes among Ghanaian adults: The RODAM Study

Ina Danquah (Dr. rer. medic.)^a,b,†,*^, Juliet Addo (PhD)^c,†^, Daniel Boateng (PhD)^d,e^, Kerstin Klipstein-Grobusch (PhD)^d,e^, Karlijn Meeks (PhD)^f^, Cecilia Galbete (PhD)^a^, Erik Beune (PhD)^f^, Silver Bahendeka (MD)^g^, Joachim Spranger (M.D.)^h^, Frank P.
Mockenhaupt (Dr. med.)^i^, Karien Stronks (PhD)^f^, Charles Agyemang (PhD)^f^, Matthias B. Schulze (Dr. P.H.)^a^, Liam Smeeth (PhD)^c^

Supplemental Figure S1: Flow-chart of multiple imputed data and for exclusions of participants with implausible data

Assessed for eligibility (n = 6,385)

## Analysis

## Exclusions

- Outside age range: 25-70 yrs (10; 2%)
- Extreme values for leg length-to-height ratio: 0.47 < LHR < 0.54 (8; 1%)
- Outside age range: 25-70 yrs (207; 4%)
- Extreme values for leg length-to-height ratio: 0.47 < LHR < 0.54 (98; 2%)

Analyzed (n = 5,052)

Analyzed (n = 523)

Missing or implausible values for:

- Waist circumference (n = 3; 0.5%)
- Exposure variables (n = 111; 21%)

Leg length and height

Father’s education and mother’s education

- Covariates (n = 249; 46%)

Participant‘s education and occupation

Energy intake

Physical activity

Smoking status

Body weight

Missing or implausible values for:

- Waist circumference (n = 17; 0.3%)
- Exposure variables (n = 1297; 24%)

Leg length and height

Father’s education and mother’s education

- Covariates (n = 2422; 45%)

Participant’s education and occupation

Energy intake

Physical activity

Smoking status

Body weight

## Type 2 diabetes status

Positive (n = 541)

- Type 2 diabetes:

Self-reported diabetes and/or fasting plasma glucose >7.0 mmol/L and/or documented anti-diabetes medication

## Multiple imputation

Negative (n = 5,357)

- No type 2 diabetes

Included (n = 5,898)

Excluded (n = 487)

- Missing physical examination
- Missing blood sample

## Recruitment

**Supplemental Table S1: General characteristics of the RODAM study population according to sex and study site**

| **Characteristics** | **Total**  **(n = 5,575)** | **Rural Ghana**  **(n = 1,004)** | **Urban Ghana**  **(n = 1,440)** | **Europe**  **(n = 3,131)** |
| --- | --- | --- | --- | --- |
| **Male** | **2,118** | **397** | **409** | **1,312** |
| Age (years) | 46.8 ± 11.1 | 46.3 ± 12.9 | 46.6 ± 11.9 | 47.1 ± 10.3 |
| Education |  |  |  |  |
| Never/elementary | 20.4% | 40.7% | 23.1% | 13.5% |
| Lower | 40.3% | 38.0% | 44.4% | 39.7% |
| Intermediate | 22.0% | 14.6% | 21.9% | 24.3% |
| Higher | 17.3% | 6.7% | 10.7% | 22.6% |
| Occupational class |  |  |  |  |
| High | 31.4% | 13.5% | 32.3% | 36.3% |
| Low | 68.6% | 86.6% | 67.7% | 63.4% |
| Body mass index (kg/m^2^) | 25.3 ± 4.4 | 20.8 ± 2.9 | 24.1 ± 3.7 | 27.0 ± 3.9 |
| Waist circumference (cm) | 88.1 ± 12.0 | 76.7 ± 8.1 | 84.7 ± 10.5 | 92.5 ± 10.8 |
| Abdominal obesity (yes) | 11.3% | 0.8% | 3.4% | 17.0% |
| Smoking (current or former) | 20.5% | 21.8% | 19.3% | 20.4% |
| Physical activity (MET-h/week) | 72 (14-160) | 104 (52-174) | 84 (24-174) | 54 (9-184) |
| Total Energy intake (kcal/day) | 2508 (1990-3229) | 2494 (2023-3077) | 2352 (1919-2787) | 2587 (2012-3380) |
| Type 2 diabetes (yes) | 11.0% | 3.8% | 11.3% | 13.1% |

| **Characteristics** | **Total**  **(n = 5,575)** | **Rural Ghana**  **(n = 1,004)** | **Urban Ghana**  **(n = 1,440)** | **Europe**  **(n = 3,131)** |
| --- | --- | --- | --- | --- |
| **Female** | **3,457** | **607** | **1,031** | **1,819** |
| Age (years) | 45.8 ± 10.6 | 46.6 ± 12.4 | 44.7 ± 11.2 | 46.2 ± 9.6 |
| Education |  |  |  |  |
| Never/elementary | 42.0% | 66.0% | 51.5% | 28.6% |
| Lower | 34.9% | 27.9% | 36.5% | 36.2% |
| Intermediate | 14.8% | 3.3% | 8.8% | 22.1% |
| Higher | 8.3% | 2.8% | 3.2% | 13.1% |
| Occupational class |  |  |  |  |
| High | 34.4% | 13.8% | 38.1% | 39.1% |
| Low | 65.6% | 86.1% | 61.9% | 60.9% |
| Body mass index (kg/m2) | 28.4 ± 5.6 | 23.6 ± 4.6 | 28.0 ± 5.4 | 30.3 ± 5.0 |
| Waist circumference (cm) | 92.3 ± 12.7 | 83.7 ± 11.3 | 91.2 ± 11.9 | 95.9 ± 12.1 |
| Abdominal obesity (yes) | 62.1% | 30.9% | 58.7% | 74.5% |
| Smoking (current or former) | 4.0% | 1.1% | 2.2% | 6.0% |
| Physical activity (MET-h/week) | 48 (8-132) | 70 (22-140) | 47 (2-144) | 40 (8-124) |
| Total Energy intake (kcal/day) | 2340 (1871-3002) | 2459 (1997-3066) | 2191 (1802-2683) | 2448 (1869-3177) |
| Type 2 diabetes (yes) | 8.4% | 5.4% | 8.4% | 9.4% |

Data are presented as mean ± standard deviation or as median (interquartile range) and as percentage.

**Supplemental Table S2: Associations of leg length and leg length-to-height ratio with waist circumference and type 2 diabetes by sex and study site**

|  | **Waist circumference** | | | | **Type 2 diabetes** | | | |
| --- | --- | --- | --- | --- | --- | --- | --- | --- |
| **Stratum** | **β per 1 SD decrease leg length (95% CI)** | **p** | **β per 1 SD decrease leg length-to-height ratio (95% CI)** | **p** | **OR per 1 SD decrease leg length (95% CI)** | **p** | **OR per 1 SD decrease leg length-to-height ratio  (95% CI)** | **p** |
| **Men** |  |  |  |  |  |  |  |  |
| Europe |  |  |  |  | 172 cases/1140 non-cases |  | 172 cases/1140 non-cases |  |
| Model 1 | -0.7 (-1.3, -0.1) | 0.019 | 0.9 (0.4, 1.5) | 0.0009 | 0.95 (0.80, 1.12) | 0.517 | 1.04 (0.89, 1.23) | 0.600 |
| Model 2 | -0.7 (-1.3, -0.1) | 0.017 | 0.9 (0.3, 1.4) | 0.001 | 1.09 (0.90, 1.30) | 0.378 | 1.07 (0.90, 1.27) | 0.452 |
| Model 3 | 2.2 (1.2, 3.2) | <.0001 |  |  |  |  |  |  |
| Urban Ghana |  |  |  |  | 46 cases/363 non-cases |  | 46 cases/363 non-cases |  |
| Model 1 | -1.2 (-2.2, -0.3) | 0.012 | 1.5 (0.6, 2.5) | 0.002 | 1.26 (0.91, 1.74) | 0.168 | 1.23 (0.90, 1.69) | 0.195 |
| Model 2 | -1.2 (-2.2, -0.2) | 0.015 | 1.5 (0.5, 2.5) | 0.002 | 1.33 (0.93, 1.91) | 0.115 | 1.26 (0.91, 1.75) | 0.165 |
| Model 3 | 3.7 (1.9, 5.5) | <.0001 |  |  |  |  |  |  |
| Rural Ghana |  |  |  |  | 15 cases/382 non-cases |  | 15 cases/382 non-cases |  |
| Model 1 | -1.4 (-2.1, -0.6) | 0.0005 | 0.6 (-0.3, 1.5) | 0.177 | 1.16 (0.68, 1.99) | 0.592 | 1.52 (0.84, 2.76) | 0.166 |
| Model 2 | -1.4 (-2.1, -0.6) | 0.0006 | 0.6 (-0.3, 1.5) | 0.170 | 1.45 (0.74, 2.85) | 0.277 | 1.83 ( 0.94, 3.57) | 0.078 |
| Model 3 | 1.9 (0.3, 3.6) | 0.017 |  |  |  |  |  |  |

|  | **Waist circumference** | | | | **Type 2 diabetes** | | | |
| --- | --- | --- | --- | --- | --- | --- | --- | --- |
| **Stratum** | **β per 1 SD decrease leg length (95% CI)** | **p** | **β per 1 SD decrease leg length-to-height ratio (95% CI)** | **p** | **OR per 1 SD decrease leg length (95% CI)** | **p** | **OR per 1 SD decrease leg length-to-height ratio  (95% CI)** | **p** |
| **Women** |  |  |  |  |  |  |  |  |
| Europe |  |  |  |  | 170 cases/1649 non-cases |  | 170 cases/1649 non-cases |  |
| Model 1 | -0.7 (-1.2, -0.1) | 0.013 | 0.8 (0.3, 1.4) | 0.003 | 0.95 (0.81, 1.12) | 0.565 | 0.99 (0.84, 1.17) | 0.917 |
| Model 2 | -0.7 (-1.3, -0.2) | 0.008 | 0.8 (0.2, 1.3) | 0.005 | 1.08 (0.90, 1.30) | 0.385 | 1.01 (0.85, 1.22) | 0.876 |
| Model 3 | 2.1 (1.1, 3.1) | <.0001 |  |  |  |  |  |  |
| Urban Ghana |  |  |  |  | 87 cases/944 non-cases |  | 87 cases/944 non-cases |  |
| Model 1 | -1.0 (-1.7, -0.3) | 0.006 | 1.2 (0.4, 2.0) | 0.003 | 0.86 (0.68, 1.07) | 0.175 | 0.84 (0.65, 1.08) | 0.177 |
| Model 2 | -1.0 (-1.7, -0.3) | 0.006 | 1.2 (0.4, 2.0) | 0.004 | 0.95 (0.74, 1.22) | 0.687 | 0.90 (0.68, 1.19) | 0.463 |
| Model 3 | 3.8 (2.4, 5.3) | <.0001 |  |  |  |  |  |  |
| Rural Ghana |  |  |  |  | 33 cases/574 non-cases |  | 33 cases/574 non-cases |  |
| Model 1 | -0.8 (-1.7, 0.1) | 0.075 | 1.3 (0.3, 2.2) | 0.012 | 0.75 (0.53, 1.05) | 0.098 | 0.83 (0.57, 1.22) | 0.341 |
| Model 2 | -0.8 (-1.7, 0.1) | 0.092 | 1.3 (0.3, 2.3) | 0.010 | 0.95 (0.64, 1.40) | 0.794 | 0.93 (0.60, 1.42) | 0.720 |
| Model 3 | 3.0 (1.3, 4.8) | 0.0009 |  |  |  |  |  |  |

For waist circumference, beta-coefficients (β), 95% confidence intervals (CIs) and p-values were calculated by linear regression. For type 2 diabetes, odds ratios, 95% CIs and p-values were calculated by logistic regression. Model 1 accounted for age (years). Model 2 was additionally adjusted for smoking (current or quit/never), physical activity (MET-hours/week), and energy intake (kcal/d), and waist circumference (cm) for logistic regressions. Model 3 was additionally adjusted for body height (cm). Model 1 accounted for age (years).

**Supplemental Table S3: Multiple-adjusted associations of parental education, leg length and leg length-to-height ratio with waist circumference and type 2 diabetes, respectively, in the complete-case analysis (n = 2,908)**

|  | **Men (n = 1,070)** | | **Women (n = 1,838)** | |
| --- | --- | --- | --- | --- |
| **Waist circumference** | **Adjusted β (95% CI)** | **p-value** | **Adjusted β (95% CI)** | **p-value** |
| **Father’s education** |  |  |  |  |
| Never/low | 4.34 (2.05, 6.65) | 0.0002 | 0.39 (-1.69, 2.48) | 0.712 |
| Intermediate | 2.95 (1.09, 4.81) | 0.002 | 0.79 (-1.01, 2.59) | 0.390 |
| Higher | Reference |  | Reference |  |
| **Mother’s education** |  |  |  |  |
| Never/low | 4.63 (1.41, 7.84) | 0.005 | -0.72 (-3.85, 2.41) | 0.650 |
| Intermediate | 2.83 (0.13, 5.52) | 0.040 | -2.08 (-4.80, 0.64) | 0.133 |
| Higher | Reference |  | Reference |  |
| **Leg length per 1 SD decrease** | 2.33 (1.24, 3.42) | <.0001 | 2.61 (1.66, 3.56) | <.0001 |
| **LHR per 1 SD decrease** | 0.60 (-0.02, 1.21) | 0.058 | 0.92 (0.36, 1.48) | 0.001 |
| **Type 2 diabetes** | **Adjusted OR (95% CI)** | **p-value** | **Adjusted OR (95% CI)** | **p-value** |
| **Father’s education** |  |  |  |  |
| Never/low | 0.48 (0.19, 1.22) | 0.125 | 2.40 (1.31, 4.38) | 0.004 |
| Intermediate | 0.90 (0.50, 1.62) | 0.720 | 1.29 (0.71, 2.34) | 0.404 |
| Higher | Reference |  | Reference |  |
| **Mother’s education** |  |  |  |  |
| Never/low | 0.53 (0.15, 1.87) | 0.323 | 2.20 (0.91, 5.37) | 0.082 |
| Intermediate | 1.21 (0.53, 2.77) | 0.656 | 1.03 (0.36, 2.97) | 0.961 |
| Higher | Reference |  | Reference |  |
| **Leg length per 1 SD decrease** | 1.11 (0.89, 1.39) | 0.344 | 0.94 (0.78, 1.14) | 0.539 |
| **LHR per 1 SD decrease** | 0.98 (0.79, 1.21) | 0.845 | 0.92 (0.76, 1.11) | 0.375 |

Beta-coefficients (β), 95% confidence intervals (CIs) and p-values for waist circumference were calculated by linear regression. The models were adjusted for age (years), study site (5 categories), smoking (current or quit/never), physical activity (MET-hours/week), and energy intake (kcal/d). Leg length was additionally adjusted for body height (cm).

Odds ratios (ORs), 95% CIs and p-values for type 2 diabetes were calculated by logistic regression. The models were adjusted for age (years), study site (5 categories), smoking (current or quit/never), physical activity (MET-hours/week), energy intake (kcal/d), body mass index (kg/m^2^), and waist circumference (cm).

**Supplemental Table S4: Associations of parental education with waist circumference adjusted for adult education and adult occupation**

| **Early-life exposures** | **Men** | | | | **Women** | | | |
| --- | --- | --- | --- | --- | --- | --- | --- | --- |
|  | **Model 2a  (β; 95% CI)** | **p** | **Model 2b  (β; 95% CI)** | **p** | **Model 2a  (β; 95% CI)** | **p** | **Model 2b  (β; 95% CI)** | **p** |
| **Father’s education** |  |  |  |  |  |  |  |  |
| Never/low | 2.3 (0.7, 3.8) | 0.005 | 2.5 (0.9, 4.0) | 0.002 | 1.1 (-0.4, 2.5) | 0.138 | 1.1 (-0.3, 2.6) | 0.111 |
| Intermediate | 1.7 (0.1, 3.3) | 0.034 | 1.9 (0.3, 3.5) | 0.020 | 1.5 (0.3, 2.8) | 0.016 | 1.6 (0.4, 2.8) | 0.009 |
| Higher | Reference |  | Reference |  | Reference |  | Reference |  |
| **Mother’s education** |  |  |  |  |  |  |  |  |
| Never/low | 1.8 (-0.1, 3.7) | 0.057 | 2.2 (0.4, 4.0) | 0.016 | 0.9 (-0.9, 2.7) | 0.326 | 1.3 (-0.6, 3.1) | 0.171 |
| Intermediate | 1.6 (-0.7, 4.0) | 0.169 | 1.8 (-0.7, 4.3) | 0.145 | -1.4 (-3.2, 0.5) | 0.143 | -1.2 (-2.9, 0.6) | 0.190 |
| Higher | Reference |  | Reference |  | Reference |  | Reference |  |

Beta coefficients (β), 95% confidence intervals (CIs) and p-values for waist circumference were calculated by linear regression, and were adjusted (aOR) for age (years), study site (5 categories), smoking (current or quit/never), physical activity (MET-hours/week), and energy intake (kcal/d).
Model 2a: further adjustment for education; Model 2b: further adjustment for occupation
